# Pancreatic ductal hyperplasia/dysplasia with obstructive chronic pancreatitis: an association with reduced pancreatic weight in type 1 diabetes

**DOI:** 10.1007/s00125-016-3867-x

**Published:** 2016-01-28

**Authors:** Tetsuro Kobayashi, Kaoru Aida, Tomoyasu Fukui, Erika Jimbo, Akira Shimada, Yasumichi Mori, Takeshi Fujii, Soroku Yagihashi

**Affiliations:** Division of Immunology and Molecular Medicine, Okinaka Memorial Institute for Medical Research, 2-2-2 Toranomon, Minato-ku, Tokyo, 105-8470 Japan; Department of Endocrinology and Metabolism, Toranomon Hospital, Tokyo, Japan; Third Department of Internal Medicine, Interdisciplinary Graduate School of Medicine and Engineering, University of Yamanashi, Yamanashi, Japan; Division of Diabetes, Metabolism and Endocrinology, Department of Medicine, Showa University School of Medicine, Tokyo, Japan; Department of Endocrinology and Diabetes, Saitama Medical University, Saitama, Japan; Department of Pathology, Toranomon Hospital, Tokyo, Japan; Department of Pathology and Molecular Medicine, Hirosaki University Graduate School of Medicine, Hirosaki, Japan

**Keywords:** Epithelial hyperplasia, GAD antibodies, LADA, Pancreas weight, Pancreatic duct, Slowly progressive IDDM, Type 1 diabetes

*To the Editor:* The report by Campbell-Thompson and colleagues [1], providing evidence that pancreatic weight is reduced irrespective of duration in type 1 diabetic donors, presented important characteristics of the pancreas in type 1 diabetes. We would like to offer two comments and introduce our own results.

First, the report by Campbell-Thompson et al [1] did not address potential differences in pancreatic weight in terms of subtypes of type 1 diabetes. Two clinical subtypes of type 1 diabetes, the classical acute/rapid-onset form (AT1D) and slowly progressive insulin-dependent diabetes mellitus (SPIDDM; now better known as latent autoimmune diabetes in adults [LADA] [2]), have been classified by a WHO consultation [3] and numerous studies, and diagnostic criteria for SPIDDM have been established by the Japan Diabetes Society (JDS) [4]. In the Campbell-Thompson study [1] the data on the two type 1 diabetes subtypes were potentially conflated with the result that data on pancreatic weight became less specific. We previously reported that the pancreases of patients with SPIDDM [5, 6] had a high frequency of reduced pancreas size and characteristic pancreatic ductal changes (cystic lesions or dilation and narrowing of pancreatic ducts), both of which are associated with pancreatic weight, while the pancreases of patients with classical AT1D did not [6].

Second, the Campbell-Thompson paper [1] argued that pancreases with reduced weight had lobular differences; this is related to currently disputed issues including lobular atrophy and exocrine inflammation in type 1 diabetic donor pancreases [7, 8]. Campbell-Thompson’s report [1] prompted us to study not only pancreatic weight but also pancreatic ductal changes, which are closely related to pancreatic lobular changes, in autopsied pancreases from individuals with the two subtypes of type 1 diabetes, SPIDDM and AT1D.

Eleven pancreases from autopsied SPIDDM patients (M/F, 5/6; age, 64 ± 14 years; range, 42–87 years; duration of diabetes, 13 ± 7 years; range, 0.3–24 years), ten pancreases from AT1D patients (M/F, 5/5; age, 60 ± 9 years; range, 42–74 years; duration of diabetes, 26 ± 11 years; range, 13–47 years), 15 pancreases from patients with type 2 diabetes (M/F, 11/4; age, 72 ± 14 years; range, 43–92 years; duration of diabetes, 13 ± 8 years; range, 3–28 years), and 19 pancreases from patients without diabetes (M/F, 10/9; age, 66 ± 6 years; range, 53–82 years) were reviewed. All donors were of Japanese ethnicity. Pancreases from heavy alcohol users and patients who had a history of DPP-4 inhibitor use and/or pancreatic tumours were excluded from the study. All SPIDDM cases were positive for GAD autoantibodies (mean titre, 56.9 U/ml; range, 3.4–398.0 U/ml; normal, <1.5 U/ml) and were treated with insulin. Their data met the JDS diagnostic criteria for SPIDDM [4]. Pancreatic tissue acquisition and data collection were performed between 1982 and 2014. The autopsied samples were obtained from Toranomon Hospital (Tokyo, Japan), University of Yamanashi (Chuo, Japan) and Saitama Saiseikai Hospital (Saitama, Japan). Tissues from the pancreatic body and tail were formalin-fixed and paraffin-embedded. Written informed consent was obtained from the next of kin on behalf of the autopsied patients. The institutional review boards of the University of Yamanashi and Toranomon Hospital approved all study protocols presented. Fisher’s exact test was used to evaluate the frequencies of lesions. Comparisons between groups were performed using the Kruskal–Wallis test and the Mann–Whitney *U* test. Values are expressed as mean ± SD.

Pancreatic weight in cases of SPIDDM (29.8 ± 8.2 g, range, 18.4–45.0 g, *n* = 11) was significantly lower than in cases of AT1D (42.1 ± 7.3 g, range, 30.0–59.5 g, *n* = 10, *p* < 0.0059 vs SPIDDM), type 2 diabetes (66.9 ± 19.4 g, range, 32.0–100.0 g, *n* = 15, *p* < 0.0001 vs SPIDDM) and non-diabetic controls (79.3 ± 9.3 g, range, 63.1–95.1 g, *n* = 10, *p* < 0.0002 vs SPIDDM). All SPIDDM pancreases showed extensive pancreatitis with mononuclear cell infiltration, fibrosis and lobular atrophy of the exocrine tissues (Fig. 1a). Dilation of the pancreatic duct, a sign of chronic ductal hyper-pressure, was also observed in all 11 SPIDDM pancreases (Fig. 1a). Ductal epithelial hyperplasia/dysplasia (DEHD)—tall, columnar, mucin-rich cells with nuclear atypia (Fig. 1b)—were observed in the branches and smaller ducts in 73% of SPIDDM pancreases (8/11; 95% CI, 46%, 98%), compared with 10% (1/10; 95% CI, 0%, 29%; *p* = 0.0007 vs SPIDDM) of AT1D pancreases, 13% of type 2 diabetes pancreases (2/15; 95% CI, 3%, 34%; *p* = 0.0033 vs SPIDDM) and 5% of non-diabetic controls (1/19; 95% CI, 0.2%, 28%; p = 0.0002 vs SPIDDM). The pancreatic duct in cases of SPIDDM showed a dilated lumen filled with mucus and sequestrated contents in all eight cases with DEHD lesions (Fig. 1a). Two SPIDDM pancreases that lacked DEHD in the examined pancreatic sections had mucous contents in dilated pancreatic ducts, suggesting the presence of downstream pancreatic duct obstruction and upstream occult DEHD. One SPIDDM pancreas, which was found to have a tortuous and dilated main pancreatic duct and cystic changes in the branched ducts in a previous study using endoscopic retrograde pancreatography [6] (Fig. 1c), had DEHD, lobular atrophy and dilated branched ducts with mucous contents (Fig. 1a, b).

**Fig. 1:**
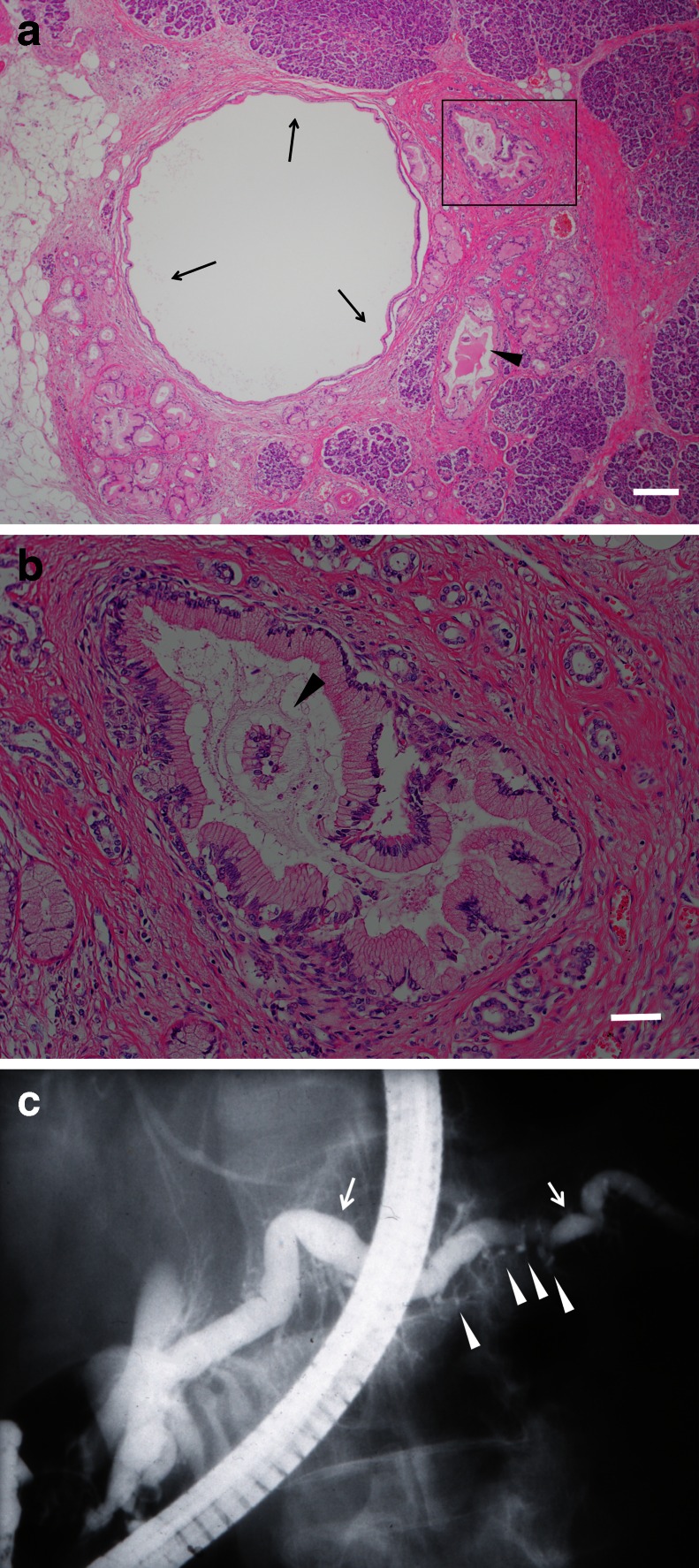
(**a**) Histological features in a pancreas from a patient with SPIDDM (woman, 76 years of age at autopsy). Marked atrophy of the exocrine pancreas, dilated pancreatic duct (arrows), pancreatic DEHD (rectangle) and mucinous contents and cell debris in the pancreatic duct (arrowhead) are shown. Haematoxylin and eosin (HE) staining, magnification ×40, scale bar, 250 μm. (**b**) Magnified view of the DEHD shown in (**a**) with nuclear atypia. The ductal lumen is filled with mucinous contents and cell debris (arrowhead), apparently causing ductal obstruction. HE staining, magnification ×200, scale bar, 50 μm. (**c**) Pancreatic ductal changes shown by an endoscopic retrograde pancreatography in the same case shown in (**a**) and (**b**). A tortuous dilated or narrow main pancreatic duct (arrows) and cystic changes in the branched ducts (arrowheads) were identified 7 years before the patient’s death

We found that pancreatic weight was significantly lower in SPIDDM cases than in AT1D cases. A higher frequency of DEHD with lobular atrophy was observed in SPIDDM than in AT1D. Suda et al [9] showed that DEHD occurs at multiple pancreatic ductal sites with highly viscous mucinous secretions and epithelial cell debris. These mucinous secretions and DEHD frequently obstruct the pancreatic branch ducts and induce obstructive chronic pancreatitis in the upstream pancreatic lobes and lobular atrophy [9]. These characteristic lesions are likely to be related to the reduced weight of the pancreas in SPIDDM and are potentially present in a proportion of patients in Campbell-Thompson’s study [1]. It may be possible that exocrine inflammation induced by DEHD-induced obstructive chronic pancreatitis initiates activation of autoreactive T cells to islet cells with subsequent destruction of beta cells through a bystander mechanism [10]. Our data had limitations due to the small sample size. Further study on the detailed immunological mechanism(s) of chronic obstructive pancreatitis-induced beta cell destruction will be needed.

## Abbreviations

AT1D: Acute-onset type 1 diabetes
DEHD: Ductal epithelial hyperplasia/dysplasia
JDS: Japan Diabetes Society
SPIDDM: Slowly progressive insulin-dependent diabetes mellitus
